# Distinguishable short-term effects of tea and water drinking on human saliva redox

**DOI:** 10.1038/s41538-024-00266-x

**Published:** 2024-04-22

**Authors:** Xiangyu Meng, Pik Han Chong, Lijing Ke, Pengwei Zhang, Li Li, Binbin Song, Zhaoshuo Yu, Pingfan Rao

**Affiliations:** 1 Food Nutrition Sciences Centre, Zhejiang Gongshang University, Hangzhou, Zhejiang 310018 China; 2 Clinical Nutrition Research Centre, Singapore Institute of Food and Biotechnology Innovation, Agency for Science, Technology and Research (A*STAR), Singapore, 117599 Singapore; 3 School of Food Science and Nutrition, University of Leeds, Leeds, LS2 9JT UK; 4 Affiliated Hospital of Hangzhou Normal University, Hangzhou Normal University, Hangzhou, 310015 China; 5 Clinical Medicine College, Hangzhou Normal University, Hangzhou, China; 6 National Nutrition Surveillance Centre, University College Dublin, Dublin, Ireland; 7 Food for Health Ireland, UCD Institute of Food and Health, University College Dublin, Belfield, Dublin 4 Ireland

**Keywords:** Health care, Physiology

## Abstract

Food consumption can alter the biochemistry and redox status of human saliva, and the serving temperature of food may also play a role. The study aimed to explore the immediate (3 min) and delayed (30 min) effects of hot tea (57 ± 0.5 °C) ingestion and cold tea (8 ± 0.5 °C) ingestion on the salivary flow rate and salivary redox-relevant attributes. The saliva was collected from 20 healthy adults before, 3-min after and 30-min after the tea ingestion. The hot or cold deionised water at the same temperatures were used as control. The salivary flow rate and redox markers in hot tea (HBT), cold tea (CBT), hot water (HW) and cold water (CW) group were analysed and compared. The results demonstrated that neither the black tea nor the water altered the salivary flow rate; the black tea immediately increased the salivary thiol (SH) and malondialdehyde (MDA) content while reduced salivary uric acid (UA) significantly. The tea ingestion showed a tendency to elevate the ferric reducing antioxidant power (FRAP) in saliva, although not significantly. The water ingestion decreased the MDA content immediately and increased the UA level significantly. Cold water was found to induce a greater delayed increase in total salivary total protein (TPC) than the hot water. In conclusion, the black tea ingestion affects the redox attributes of human saliva acutely and significantly, while the temperature of drink makes the secondary contribution.

## Introduction

Tea (*Camellia sinensis*) is the most widely consumed beverage world-wide besides water^1^. Among the major types of tea, black tea is the most popular choice, accounting for 80% of global tea consumption^2,3^. It is well-known that black tea contains natural ingredients such as polyphenols, pigments, alkaloids and polysaccharides, which are known to be beneficial for lowing the risks of various oxidative stress-induced diseases^4,5^, such as cancer, diabetes^6,7^, cardiovascular disease^1,8^ and irritated bowel diseases (IBDs)^9^. Most of the beneficial effects of tea are attributed to the antioxidant and free radical scavenging properties of its constituents, such as polyphenols and flavonoids^8,10^ and the self-assembled nanoparticles carrying these bioactive compounds^11^. These components can inhibit cancer cell proliferation, regulate glycolipid metabolism and DNA repair, enhance immune system and the functions of detoxifying enzyme^12–14^.

During the oral ingestion of tea, saliva is the first biological fluid that comes into contact with tea components^15^. Saliva plays an important role in oral redox homeostasis. In addition to immunoglobulins, antimicrobial enzymes, and growth factors, saliva contains antioxidants and is the first line of defence against oxidative stress^16,17^, possibly caused by smoking, alcohol consumption, microorganisms, and unhealthy food intake^16,18^. The salivary antioxidants includes catalase, superoxide dismutase, glutathione peroxidase, transferrin, albumin, uric acid, vitamin C, etc.^16,19,20^. Thiols containing sulfhydryl groups also act as antioxidants in saliva^21^. The surface-exposed cysteine residues of proteins are particularly sensitive to oxidation by almost all forms of reactive oxygen species (ROS), and the oxidation of these sulphur-containing amino acid residues is reversible^22^. These proteins therefore serve as antioxidants^23^. In addition, as a product of lipid oxidation, malondialdehyde levels are often used as an indicator of oxidative stress in saliva^19,20^.

Salivary flow rate has to be considered when studying saliva due to the variations existed among individual, ranging from approximately 0.3 to 0.4 mL/min and the composition of saliva is highly dependent on it^24^.

Most of the previous studies have highlighted the beneficial effects of consuming tea in the long term. In this study, we focused on the immediate effects (3 min after) and delayed effects (30 min after) of black tea consumption on the biochemical attributes of saliva, which are short-term effects and closely related to oral sensation and potentially mucosal inflammation. Further, temperature of tea drink is an important factor affecting the perception and physiological influences of tea. In the past, extensive attention has been paid to the effects of fermentation temperature and brewing temperature on the quality and sensory profiles of black tea^3,25^. However, as beverages, hot tea and cold tea bring different oral sensations, and the effect of tasting temperature on salivary composition and redox state has received little attention. In one report, water at 3 °C stimulates an increase in salivary flow rate more than water at other temperatures (13 °C, 23 °C, and 33 °C), which changes the content of other substances in the salivary composition^26^. Therefore, the effect of black tea temperature on saliva is worth paying attention to.

Previous work in the laboratory has investigated the influences of consuming the vine tea, oolong tea and black tea on the salivary compositions^15,24^. However, the possible influences of water in these tea infusions have not been ruled out. In this study, the drinking water at the corresponding temperature of black tea (Lapsang souchong) infusion was used as control to observe the changes in salivary flow rate, total protein content, total antioxidant capacity, uric acid concentration, thiol content and malondialdehyde content before, 3-min after and 30-min after the black tea (water) consumption. The study is expected to distinguish the influences of the tea components and water in the black tea infusion on the redox status of healthy adults’ saliva, as well as to illuminate the potential effects of tea drinking temperature.

## Results and discussion

### Salivary flow rate (SFR)

**Fig. 1:**
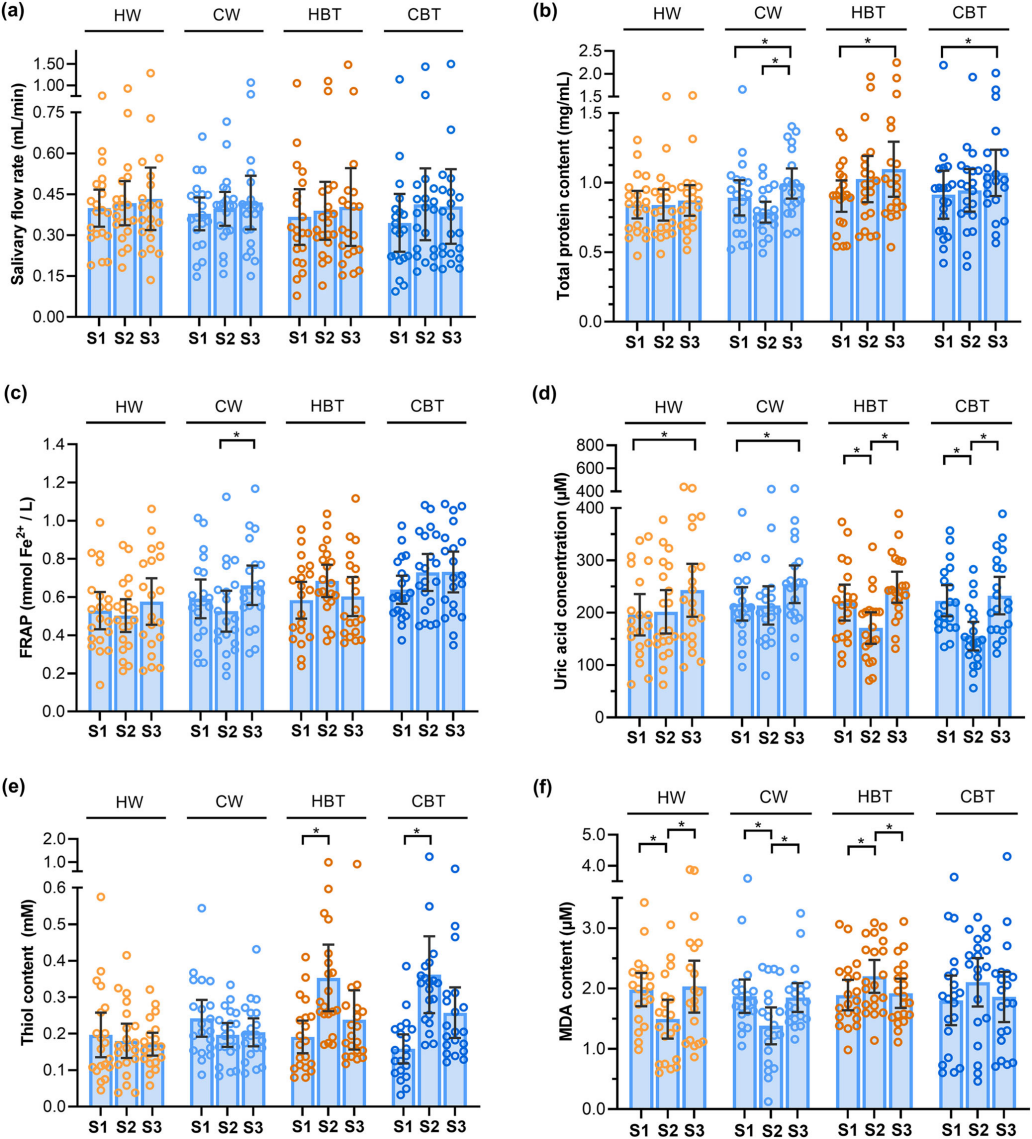
The change of salivary. **a** flow rate, (**b**) total protein, (**c**) antioxidant capacity, (**d**) uric acid, (**e**) thiol content and (**f**) MDA content after consuming different samples. ‘*’ indicates significant differences in means ± SD between different stages (*p* < 0.05). Tea samples consumed before, after and after 30 min are denoted as Stage 1, Stage 2 and Stage 3, respectively, and are represented by S1, S2 and S3. Consumption of the same sample (black tea or water) was repeated for two days, and saliva from the same volunteer for two days was evenly mixed at the time of the assay.

As shown in Fig. 1a and Table 1, there were no significant changes in salivary flow rates of the resting status (S1), 3-min (S2) and 30-min (S3) after drinking water or black tea (*p* > 0.05), although the SFR increased mildly after drinking both liquids. It indicates neither tea nor water significantly stimulates the salivary secretion. The maximum SFR was found 30-min after ingestion in all groups. The highest average SFR (0.433 mL/min) was observed in assessors drinking hot water, while the highest individual SFR (1.499 mL/min) was found in an assessor drinking the cold tea. Within each testing group/stage, a large individual difference was found, which is consistent with previous reports^15,27^. Some participants constantly showed either much higher or much lower SFRs than the average, no matter what sample they drank. For example, volunteer No.10 and No.17 always reported the lowest SFRs, while volunteer No.13 and No.20 always showed the highest.

**Table 1:** The salivary flow rate, total protein content, antioxidant capacity, uric acid, thiol and MDA content after consuming hot/cold water or black tea

Sample	SFR	TPC	FRAP	UA	SH	MDA
Unit	mL/min	mg/mL	mmol Fe^2+^/L	µM	µM	µM
Stage 1						
HW	0.399 ± 0.064^a^	0.843 ± 0.093^a^	0.529 ± 0.091^b^	196.2 ± 37.1^a^	196.7 ± 57.1^a^	1.981 ± 0.258^a^
CW	0.378 ± 0.056^a^	0.891 ± 0.119^a^	0.590 ± 0.095^a,b^	216.8 ± 29.8^a^	242.1 ± 106.4^a^	1.872 ± 0.259^a^
HBT	0.366 ± 0.096^a^	0.904 ± 0.105^a^	0.583 ± 0.091^a,b^	219.5 ± 32.2^a^	191.4 ± 103.5^a^	1.891 ± 0.236^a^
CBT	0.345 ± 0.099^a^	0.914 ± 0.162^a^	0.639 ± 0.069^a^	222.8 ± 27.9^a^	158.3 ± 37.2^a^	1.801 ± 0.385^a^
Stage 2						
HW	0.417 ± 0.076^a^	0.840 ± 0.105^b,c^	0.503 ± 0.081^b^	201.7 ± 38.8^a,b^	180.4 ± 43.8^b^	1.491 ± 0.302^b^
CW	0.396 ± 0.058^a^	0.788 ± 0.070^c^	0.526 ± 0.100^b^	213.9 ± 34.3^a^	196.6 ± 83.0^b^	1.381 ± 0.287^b^
HBT	0.389 ± 0.099^a^	1.026 ± 0.155^a^	0.685 ± 0.080^a^	170.8 ± 28.1^b^	352.9 ± 160.0^a^	2.201 ± 0.255^a^
CBT	0.413 ± 0.123^a^	0.946 ± 0.143^a,b^	0.729 ± 0.090^a^	155.2 ± 25.5^b^	361.6 ± 178.7^a^	2.102 ± 0.374^a^
Stage 3						
HW	0.433 ± 0.107^a^	0.872 ± 0.103^b^	0.577 ± 0.114^b^	243.0 ± 47.2^a^	171.4 ± 29.2^a^	2.031 ± 0.402^a^
CW	0.420 ± 0.092^a^	0.994 ± 0.102^a^	0.661 ± 0.097^a,b^	254.3 ± 33.7^a^	208.7 ± 92.5^a^	1.849 ± 0.225^a^
HBT	0.403 ± 0.133^a^	1.097 ± 0.185^a^	0.602 ± 0.096^a,b^	248.8 ± 27.8^a^	237.7 ± 156.4^a^	1.920 ± 0.228^a^
CBT	0.405 ± 0.128^a^	1.071 ± 0.156^a^	0.732 ± 0.100^a^	232.7 ± 33.4^a^	257.4 ± 138.8^a^	1.859 ± 0.390^a^

The values were expressed as means ± SD. Tea samples consumed before, 3-min after and 30-min after are denoted as Stage 1, Stage 2 and Stage 3, respectively. ^a, b, c^ indicates significant differences at the same stage.

In general, the unstimulated whole salivary flow rate ranges from 0.25 to 0.90 mL/min with a mean value of approximately 0.4 mL/min, which could be stimulated by vision, smell or taste or parasympathetic nerve activity occurs^28,29^. The mean value of resting SFR was 0.384 mL/min in this study, which is consistent with the previous studies. However, a greater number of maximum SFR was reported here. It is known that the SFR is correlated to the size of the individual’s salivary glands, which is particularly pronounced in stimulated SFR^27^. In addition, the SFR of female volunteers might be elevated during their the menstrual cycle^30^. The reason why we are observing a relatively big variation between individuals in this study remains unclarified. The greater volume or higher concentrations of black tea infusion, the different methods or timing of saliva collection, and the longer period of drink intervention may be needed to further verify the above observations.

Usually, chewing or drinking food can cause an increase in salivary flow rate. Consumption of solid foods and chewing show the more dominant effects on salivary secretion than the beverages and drinking^27^. The gum chewing increases SFR by 2-5 times^31^. Our results demonstrate that neither water nor the black tea infusion immediately promote the saliva secretion in healthy adults.

In addition, the temperature of oral exposure may affect saliva secretion. The transient membrane potential cation receptor melatonin 8 (TRPM8) is a cold receptor in the oral cavity that can be stimulated by cold and menthol^32^, and the stimulation of cold receptors leads to increased salivary flow rate^33^. As reported by Lee et al., the parotid gland saliva flow was stimulated at 10 °C, but not at 22 °C and 44 °C^34^. Brunstrom et al. found that cold water (at 3 °C) significantly increased salivary flow rate compared to the warmer water (at 13 °C, 23 °C and 33 °C)^26^. The cold water (at 8 °C) didn’t induce a higher SFR than the hot water (at 57 °C), which could be attributed to the wider time gap between drinking and saliva collection than the Brunstrom’s report (after 3-min or 30-min v.s. immediately after ingestion), or to the different method of saliva collection (spontaneous saliva flow from the whole oral cavity v.s. a cotton swab sit underneath the tongue).

### Total protein content of saliva (TPC)

The influence of ingesting different samples on the total protein content of saliva is presented in Fig. 1b and Table 1. Salivary TPC ranged from 0.39 to 2.26 mg/mL. The TPC of the tea drinkers increased right after the ingestion, although insignificantly compared to their resting level (Stage 1, *p* > 0.05), and is significantly higher than the hot water and cold water groups (*p* < 0.05), respectively. The TPC further increased 30 min after ingestion and became significant even compared to Stage 1 (*p* < 0.05). Moreover, the salivary protein level slightly decreased 3 min after cold water ingestion (*p* > 0.05) and significantly increased 30-min after cold water ingestion (*p* < 0.05). The ingestion of hot water did not change the TPC.

The secretion of protein and fluid from salivary gland is regulated by parasympathetic nerve and sympathetic nerve impulses. The parasympathetic nerve impulses produce high-flow and low-protein saliva, while the sympathetic nerve impulses produce low-flow and high-protein saliva^35^. As shown in Figs. 1a, b, the rather similar SFR in all groups and the significantly elevated TPC in the tea ingestion group indicates the black tea ingestion may possess more potent impacts on the sympathetic nerves. The high salivary TPC accompanied by low SFR was observed in assessor No.8/10/12. Assessor No.20, however, exhibited low salivary TPC and high SFR. In addition, the proteins expressed by different salivary glands vary greatly^36^. For example, the serous fluid from parotid gland does not contain mucin but is rich in amylase and proline-rich proteins (PRP). Black tea contains rich number of tannins, which readily bind to salivary proline-rich proteins. As a result, the anti-nutritional effects of tannins, such as growth retardation and impaired nutrition absorption^37^, could be mitigated. The protein compositions of saliva after tea ingestion need to be further studied to reveal their salivary glands origin.

The study showed a relatively stronger promotion in TPC by ingesting the hot tea than the cold tea, while the hot water exhibited the weaker influences on TPC than the cold water. In Eccles’ study, the cold stimulation (temperature) to the oral receptors leads to elevated saliva secretion^38^ and a higher level of salivary proteins. It is known that the whole-body and continuous exposure to cold environment (i.e., at 4 °C for 15 min) significantly increases the salivary flow rates and protein concentration^39^, whilst in this study, a short (in seconds) and partial (mouth only) exposure to cold drink (at 8 °C) only induced a mild increase in the salivary proteins and no significant changes in the saliva secretion.

### Total antioxidant capacity (FRAP)

As shown in Fig. 1c and Table 1, the black tea ingestion tended to increase the FRAP, which was significantly higher than that of water ingestion (*p* < 0.05) but not significant when compared to the baseline levels (Stage 1, *p* > 0.05). The FRAP of cold tea ingestion remained at the same level 30 min after, indicating a longer influence than the hot tea. The salivary FRAP seemed to be reduced immediate after drinking hot water and cold water, although insignificantly (*p* > 0.05) and resumed to the initiate level by hot water (*p* > 0.05) or a significantly higher level by cold water (*p* < 0.05). The delayed effect of cold-water (30-min after ingestion) on FRAP echoed well with the effect of cold black tea ingestion, implying the cold drink may possess more profound impacts on salivary FRAP than the hot drink.

The effects of beverages on human saliva FRAP was much less reported than the blood plasma. Villaño et al. found that ingestion of 500 mL of oolong beverage contained oolong tea extract (2.4 g/L) significantly increased plasma FRAP in healthy subjects within 1 h to 4 h^40^. The study of Wiseman et al. and Langley also found that drinking green tea and black tea increased the plasma FRAP of subjects, respectively^41,42^. Azimi et al. found that drinking green tea effectively boosted the antioxidant capacity of smokers’ saliva in a short run (7 days) and a long term (3 weeks)^43^. Chong et al. reported the acute changes caused by tea drinks: drinking an oolong tea (Tie-Guan-Yin) and a black tea did not significantly change the salivary FRAP, while intake of vine tea enhanced the antioxidant capacity of saliva over a longer period of time, which were related to the high level of polyphenols and flavonoids in vine tea^24^. This may be a profound report on the immediate and short-term changes in the reducing power of saliva caused by tea drink, which demonstrates the salivary FRAP could change in response to black tea in a matter of minutes.

### Salivary uric acid concentration (UA)

As shown in Fig. 1d and Table 1, the black tea ingestion, either hot or cold, significantly reduced the salivary uric acid level 3 min after the ingestion (*p* < 0.05), which rose back to the initiate level after 30 min. The temperature of tea soup had little influence. Both hot water (58 °C, 200 mL) and cold water (7 °C, 200 mL) significantly increased salivary uric acid levels (*p* < 0.05) 30 min after the water ingestion. The results were in line with the previous studies reported by Chong et al.^24^. The salivary UA level in this study remained in the normal range of healthy adults.

Uric acid is believed to be the dominant antioxidant compound in saliva, as it accounts for about 70% of the total antioxidant capacity^44^. Recent studies confirmed a positive correlation between circulating UA and salivary UA^45,46^. However, the extraordinary high level of salivary uric acid is associated with body fat accumulation, liver steatosis and increased incidence of psychological disorders^47–49^. The external factors can rapidly alter salivary uric acid concentration. Lucas et al. reported that acute social stress rose the adults’ salivary UA level rapidly and significantly^50^. In another study, the cold pain stimulus at 5 °C rose salivary UA level in healthy young adults about half an hour after the stimulation, demonstrating a delayed response to acute physical stress^51^. As in this study, the ingestion of cold or hot water caused a delayed increase in the salivary UA levels, too.

Little was known on the effect of black tea consumption on salivary uric acid. The effects of black tea extract on plasma uric acid in mice have been highlighted in multiple studies^52,53^. The black tea extracts reduced plasma uric acid in hyperuricaemic mice to a higher extent than the green tea extracts, which is mainly due to the EGCG inhibition of xanthine oxidase (XO) and adenosine deaminase (ADA) in liver, thereby reducing uric acid synthesis^54^. On the other hand, tea catechins and theaflavin can regulate uric acid transport proteins to inhibit plasma uric acid excretion^55^. It remains unclear whether the black tea induced acute decrease in salivary UA could be attributed to the same mechanism.

### Salivary thiol content (SH)

The salivary free-thiol content indicates the level of oxidative stress^15^. As shown in Fig. 1e and Table 1, ingestion of water did not cause any significant changes in the SH content (*p* > 0.05). In comparison, regardless of the temperature of tea, the ingestion of black tea rapidly and significantly increased the SH (*p* < 0.05), which was decreased after 30 min but still stayed at the level higher than its resting level (*p* > 0.05). As the concentration of salivary thiol did not change in proportion to the total protein content, it is proposed that the free thiol (SH) may not mainly attribute to the proteins. The low molecular weight thiols may also play a part.

The low molecular weight thiols, e.g., glutathione (GSH), are crucial in many physiological processes, as in redox reactions, alkylation reactions, metal binding^56^, and mitigating oxidative stress of cells^57^. Environmental challenges like smoking can cause a decrease in salivary thiol levels^16,17^. Such decrease was also found in the patients of oral squamous cell carcinoma, oral leukoplakia^58^, Alzheimer’s disease, and many other chronic degenerative diseases^59^. Chinese black tea has been found to have preventative effects against these chronic diseases, often by effectively improving GSH and SOD levels in body fluids and mitigating oxidative damages^60,61^. The immediate increase of salivary thiol levels reported here indicates an elevated reducing power in saliva, echoing the elevated salivary FRAP and may provide a fast-responding biomarker for in vivo validation of antioxidant activity of food components. The possible role of GSH reductase in saliva is not clear and warrant a further investigation.

### Salivary malondialdehyde content (MDA)

Oxidative stress causes lipid peroxidation and induces the formation of various products, including malondialdehyde^62,63^. As shown in Fig. 1f and Table 1, drinking hot black tea immediately elevated the salivary MDA level by 16.4% (S2, *p* < 0.05) to 2.20 µM, while the cold tea showed the same tendency but not significantly. Both hot-water and cold-water ingestion suppressed the salivary MDA significantly by 24.7% and 26.2%, respectively (*p* < 0.05), demonstrating neither temperature nor water fraction have dominant effects on salivary MDA. Black tea ingestion mildly increased the salivary MDA level, which could be attributed to the significant decrease in UA and therefore a weaker reducing power. The increase in SH only partially compensated the loss of reducing power.

In general, the extraordinary high levels of salivary MDA are often associated with oral inflammation and metabolic diseases^64–66^. The salivary MDA content of healthy individuals aged 20-39 was reported to be 1.87 ± 0.68 µM^67^, which is consistent with our results. Freese et al. found that green tea extract significantly reduced plasma malondialdehyde (MDA) concentration of healthy females^68^. The long-term effects of black tea on salivary MDA have rarely been studied. Sun et al. found that the black tea polysaccharide significantly mitigated the CCl_4_-induced increase in liver MDA in mice^60^. As reported previously, the same kind of black tea increased the salivary MDA^15^, while the oolong tea and vine tea took longer time (30 min) to raise the salivary MDA level^24^. The MDA increments caused by the tea ingestion is temporary and well below the pathological increments. It becomes a curious but maybe meaningful subject how the black tea will affect the salivary MDA of patients of oral inflammation and metabolic diseases. Furthermore, the relation between the immediate and long-term changes of salivary MDA in response to black tea ingestion would help us to better understand the human physiological influences of tea, thus needs to be clarified by further studies.

### Correlation analysis

**Fig. 2:**
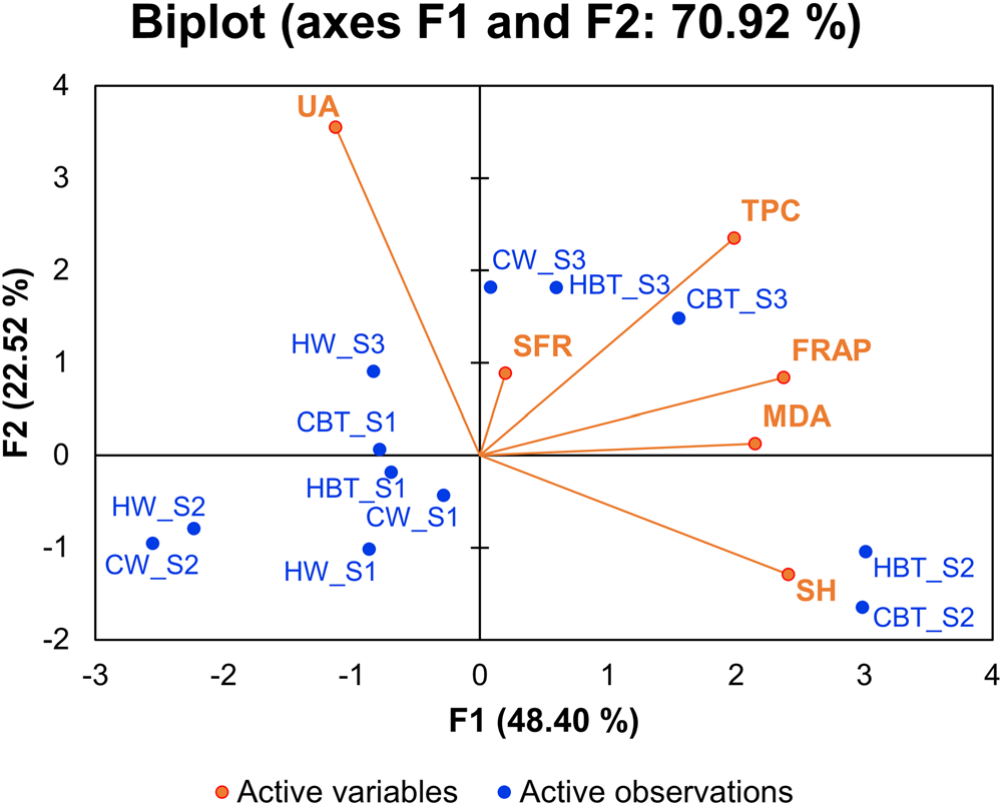
Principal component analysis for correlations among the salivary parameters. HW (Hot water), CW (Cold water), HBT (Hot black tea) and CBT (Cold black tea) represents the sample variety and stage 1, 2 and 3 are expressed by S1, S2 and S3. Salivary flow rate, total protein content, ferric reducing antioxidant power (FRAP), uric acid concentration, thiol content, and malondialdehyde content are denoted as SFR, TPC, FRAP, UA, SH and MDA, respectively.

Pearson correlation and principal component analysis were performed to reveal the correlation of the six salivary attributes in 20 subjects. Table 2 shows the factor loadings for the analysed variables from F1 to F5. A biplot of the principal components (F1 and F2) was composed to demonstrate the relationships among the variables, as shown in Fig. 2. Component 1 and 2 were used to explain 70.92% of total variation of the data set. The large loadings of TPC, FRAP, SH and MDA contribute to F1, while F2 is attributed to UA and partially TPC, and F3 is mainly attributed to SFR. The SFR has a very little loading in F1 and F2. The variables (orange lines) were distributed in three quadrants: SFR, TPC, FRAP, and MDA in quadrant 1; SH in quadrant 2; UA in quadrant 4. SH was negatively correlated with UA.

**Table 2:** Factor loadings of the analytical variables

	F1	F2	F3	F4	F5
SFR	0.005	0.050	0.942	0.000	0.002
TPC	**0.538**	0.351	0.015	0.021	0.066
FRAP	**0.768**	0.045	0.021	0.045	0.121
UA	0.173	**0.800**	0.005	0.003	0.001
SH	**0.792**	0.106	0.023	0.035	0.018
MDA	**0.629**	0.001	0.001	0.369	0.000

The bold value indicates that the variable accounts for the majority of the factor loadings.

The distribution of observations (blue dots) was shown in the biplot. The objects at stage 1 (before ingestion of water or tea, labelled as ‘XXX_S1’) stayed close to each other and mainly in quadrant 3 except CBT-S1 fell just across the border to quadrant 4, while the objects of hot water or cold water at stage 2 (HW-S2 and CW-S2) distributed in quadrant 3, and staying close to each other but away from the objects of stage1. All these six objects are not related to the variables. It indicates that drinking water, either hot or cold, is unlikely to cause immediate and significant changes in the six salivary attributes. The two objects of hot tea and cold tea at stage 2 distributed in quadrant 2 while the objects of tea and water at stage 3 were distributed in quadrant 1, except the object of hot water in quadrant 4. It suggests the immediate impacts of black tea consumption on saliva are distinguishable from the salivary attributes of resting status and 30 min after the drink. The black tea consumption, both hot and old, increased the level of salivary thiol while decrease that of uric acid.

**Fig. 3:**
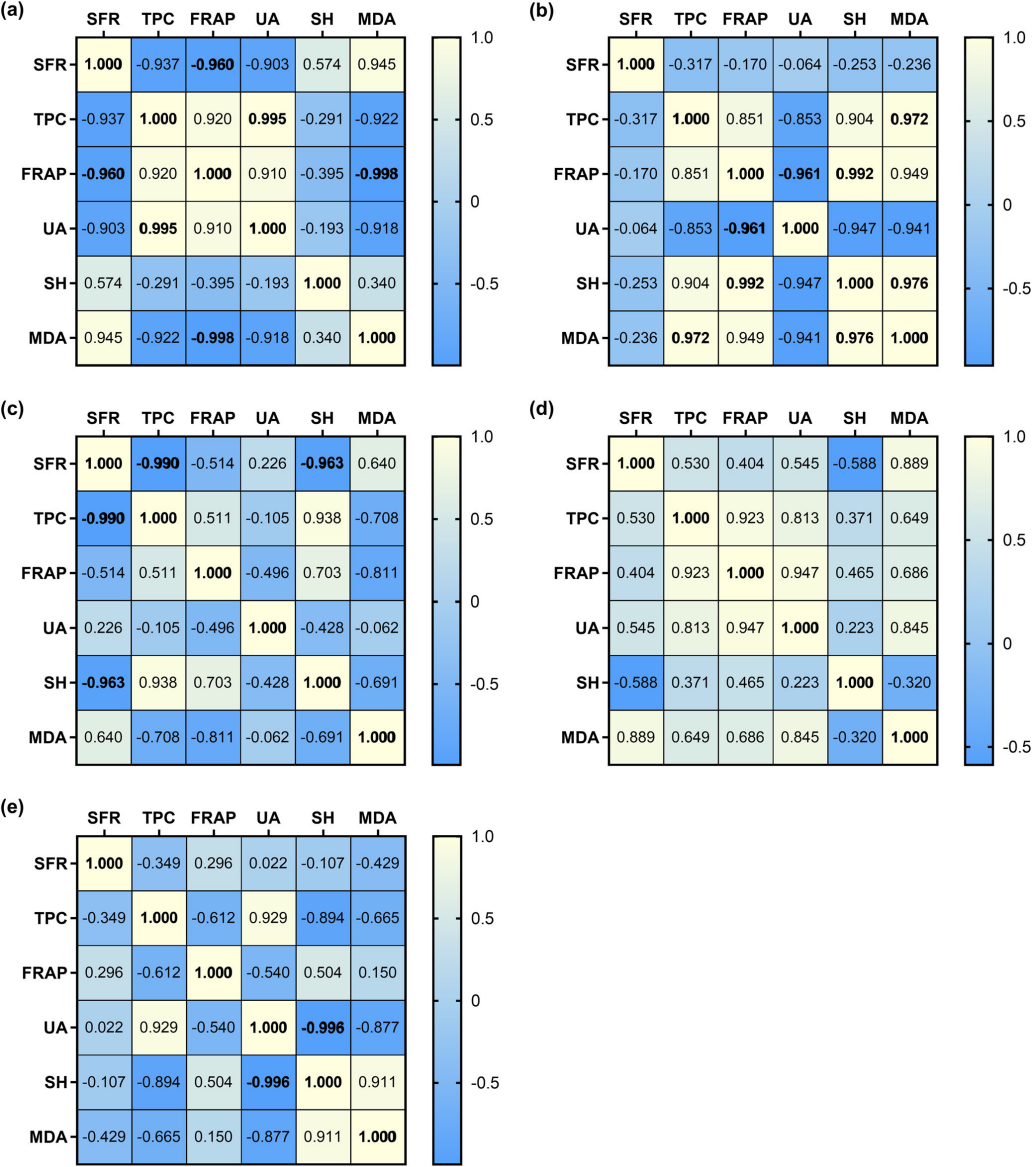
Correlation coefficient of the analytical variables of saliva. **a** Before drinking black tea and water, S1. **b** 3 min after the black tea and water ingestion, S2. **c** 30 min after the black tea and water ingestion, S3. **d** 3 min-after v.s. 30 min-after water ingestion. **e** 3 min-after v.s. 30 min-after black tea ingestion.

The correlations among salivary parameters at different stages were analysed by Pearson’s correlation and correlation coefficients were presented in Fig. 3. The values marked in bold indicate a high statistical correlation between the two variables. The positive or negative correlation were marked in yellow or blue, respectively. Before ingestion of water or tea (Stage 1) (Fig. 3a), a strong positive correlation was clearly shown between TPC and UA (*p* = 0.005, *r* = 0.995), while the strong negative correlation was shown between SFR and FRAP (*p* = 0.040, *r* = −0.960), as well as MDA and FRAP (*p* = 0.002, *r* = −0.998). Three minutes after ingestion (Stage 2) (Fig. 3b), correlations between attributes except SFR were altered quite significantly: the strong negative correlations were found between UA and FRAP (*p* = 0.039, *r* = −0.961), and between UA and SH (*p* = 0.053, *r* = −0.947), UA and MDA (*p* = 0.059, *r* = −0.941); SH was highly and positively correlated with FRAP (*p* = 0.008, *r* = 0.992) and MDA (*p* = 0.024, *r* = 0.976); there was a significant positive correlation between MDA and TPC (*p* = 0.028, *r* = 0.972), and also FRAP (*p* = 0.051, *r* = 0.949). As shown in Fig. 3c, thirty minutes after ingestion (Stage 3), there is no significant correlation between parameters except for the strong negative correlation between TPC and SFR (*p* = 0.010, *r* = −0.990) and between SH and SFR (*p* = 0.035, r = −0.965). The strong correlations observed about half an hour ago (Stage 2) either weakened or vanished, indicating the differences between the immediate and delayed effects of water/black tea consumption are worth noting.

In addition, the correlation between salivary attributes of 3-min after and 30-min after drinking water (Fig. 3d) or black tea (Fig. 3e) was compared to demonstrate the different physiological influences of water and tea ingestion. The water ingestion generally showed a more positive but less strong correlation between all variables, comparing to Fig. 3d, e, particularly evidenced by the weaker correlation coefficient between SH and UA (*p* = 0.777, *r* = 0.223 in water versus *p* = 0.004, *r* = −0.996 in black tea), FRAP and UA (*p* = 0.187, *r* = 0.947 in water versus *p* = 0.460, *r* = −0.540 in black tea), MDA and UA (*p* = 0.155, *r* = 0.845 in water versus *p* = 0.123, *r* = −0.877 in black tea). This is in good agreement with the significant increase in salivary SH and MDA and a significant decrease in UA caused by the tea ingestion.

The Chinese tea and its components (e.g., polyphenols) have been reported to reduce the level of uric acid in vitro and in vivo, either by inhibiting xanthine oxidase and subsequently reducing the uric acid production^69^ or by modulating the expression of renal urate transporters and promoting uric acid excretion in mice^70^. However, the clinical significance of tea’s impacts on the serum UA remains debatable: according to a meta-analysis of seven observational studies on the tea consumption and the serum uric acid level, no association was found between the two^71^; a survey carried out in 2007 reported that coffee consumption was associated with lower level of serum UA and less incidence of hyperuricemia, while tea consumption was not^72^. Coordinatingly, the effects of coffee were attribute to its components other than caffeine. On reviewing the conflicting results, the possible interference of some other dietary factors like milk and sweeteners shall be brought into consideration, which have been found to compromise the antioxidant capacity of green tea and black tea in animal model studies^73^.

The acute and short-term influences of black tea or water ingestion on several physiological attributes of saliva were assessed in this study. In summary, neither the black tea nor the water altered the salivary flow rate. The black tea affects the redox attributes of human saliva acutely and significantly, which are distinguishable from the effects of water.

Regarding the immediate effects after ingestion, the black tea significantly increased the salivary thiol and MDA levels and decreased the salivary uric acid, while water decreased the MDA. When it comes to the delayed effect, the black tea ingestion significantly increased the salivary TPC, while water increased the salivary uric acid significantly. The tea ingestion showed a tendency to elevate the ferric reducing antioxidant power (FRAP) in saliva, although not significantly. The temperature of drink also affects the salivary attributes. Cold water was found to induce a greater delayed increase in total salivary total protein (TPC) than the hot water.

The study helps to gain a better understanding of the short-term influences of beverage on human oral physiology, which may affect the sensory perception and digestion of food. It provides meaningful data and useful methods for future studies, while demonstrating the alternative influences of the drink’s temperature on the biochemical attributes of saliva. Further studies are warranted to illuminate the molecular and biological mechanism of the black tea’s regulating effects on salivary redox, as well as its correlation to the long-term influences on health.

## Methods

### Materials

The black tea (Lapsang souchong) was purchased from Wuyi Mountain Zhengshan Tea Industry Co., Ltd, China. The 2,2ʹ-Biquinoline-4,4ʹ-dicarboxylic acid (BCA), sodium tartrate dibasic dihydrate, sodium carbonate, sodium hydroxide, sodium bicarbonate, hydrochloric acid, copper (II) sulphate pentahydrate, disodium hydrogen phosphate dodecahydrate, Sodium dihydrogen phosphate dihydrate, 4-Aminoantipyrine, potassium ferricyanide trihydrate, sodium 3,5-dichloro-2-hydroxybenzenesulfonate, Triton X-100, Lithium carbonate, Sodium acetate, acetic acid, iron (III) chloride hexahydrate, 2,4,6-Tris(2-pyridyl)-s-triazine (TPTZ), iron (II) sulphate heptahydrate and trichloroacetic acid (TCA) were obtained from Sinopharm Chemical Reagent Co., Ltd, Shanghai, China. The bovine serum protein (BSA), peroxidase from horseradish, ascorbate oxidase from Cucurbita sp., urease from Candida sp., uric acid, 5,5ʹ- dithiobis-(2-nitrobenzoic acid) (DTNB), Tris(hydroxymethyl)aminomethane (Tris), L-cysteine, thiobarbituric acid (TBA), 1,1,3,3-Tetraethoxypropane (TEP) were purchased from Sigma-Aldrich Co. Ltd, Shanghai, China.

### Participants recruitment and ethical arrangements

The study was performed at the Food Nutrition Sciences Centre at Zhejiang Gongshang University. Twenty healthy volunteers with age between 20 and 30 years old (13 females and 7 males, BMI range from 17.98–24.03) participated in this experiment. They were in good health, did not have smoking, drinking and tea consumption habits, did not have any incidents of oral diseases or surgery (such as wisdom teeth extraction, orthodontic treatment, mouth ulcers, etc.) in the past 3 months. Participants with any history of systemic disease, regular use of mouthwashes, medications or vitamin supplements in the past 3 months, and excess weight were excluded from the study.

Subjects were well informed with the purpose and protocol of the study and signed the informed consents forms prior to their participating in the tea/water ingestion and saliva collection. This study on human saliva collection was approved by the University Ethics Committee, Zhejiang Gongshang University, reference number 2021-13.

### Preparation of tea infusion

For preparation of the black tea infusion, the ratio of black tea leaves (g) to boiling deionised water (mL) was set to 1:30, according to the local tea drinking custom in Wuyi mountain, Fujian, China. Each portion of tea leaves is steeped twice, 15 s each time. Although the tea/water ratio is high, the resulting tea infusion taste quite pleasant due to the rather short steeping time. The steeping and separation of tea leaves and tea infusion are carried out using a press filtration teapot. According to previous research^24^, the chemical composition and antioxidant activity of Lapsang souchong black tea are shown in Table 3. For hot tea, the tea infusion was kept in a thermal flask (Thermos, Japan) to ensure the drinking temperature to be 57 ± 0.5 °C. For cold tea, the tea infusion was cooled down to room temperature and kept in the refrigerator until ingestion, to ensure the drinking temperature to be 8 ± 0.5 °C. During each saliva collection, participants were asked to drink 200 mL of tea or water, respectively.

**Table 3:** The chemical compositions and antioxidant activity of Lapsang souchong black tea

Sample	Total polyphenols (µg GA/mL)	Total Flavonoids (µg DMY/mL)	Total Polysaccharide (µg/mL)	Total Protein (µg/mL)	Free Amino Acids (µg/mL)	FRAP (mmol Fe^2+^/L)
Lapsang souchong	106.8 ± 14.1	44.0 ± 4.2	210.2 ± 11.9	146.3 ± 13.5	46.2 ± 0.3	1.3 ± 0.1

Values displayed represent the means with 95% confidence interval. Means without the same letter indicate significant difference in particular parameter. This table is adapted from a previous report^24^, as the tea and brewing method are exactly the same as this study.

*DMY* dihydromyricetin, *GA* gallic acid.

### Saliva collection

To be in line with the previous studies, saliva collection was divided into 3 stages, before, 3-min after and 30-min after drinking samples (HW, CW, HBT and CBT), denoted as stage 1, stage 2 and stage 3, respectively. Saliva collection was performed daily between 8:30 am and 11:00 am. Participants were asked to tilt their heads slightly and to reduce facial and oral movements to passively flow saliva from the mouth into a sterile collector for 10 min, denoted as stage 1 (S1, baseline); after a 20-min rest, they were asked to drink black tea within 2 min and rest for 3 min before continuing saliva collection for another 10 min, denoted as stage 2 (S2, immediate effect of tea drinking). To avoid errors caused by the residue of black tea in the mouth, discard the saliva collected in the first 30 s. For stage 3 (S3), saliva was collected over a 10 min period starting from 30 min after drinking the tea. The collected saliva was immediately centrifuged at 4 °C for 15,000 g for 30 min to remove the remaining residue and cellular debris. After centrifugation the supernatant was transferred to a centrifuge tube and stored at −80 °C until analysed. In this study, consumption of the same sample (black tea or water) and collection of saliva sample was conducted twice on two separated days. The saliva from the same volunteer was evenly mixed prior to the determination of biochemical attributes.

### Salivary flow rate (SFR)

The rate of saliva secretion by the salivary glands per unit time was taken as the salivary flow rate, which can be expressed by dividing the total volume of collected saliva by the collection time. Since more than 99% of the saliva composition is water^74,75^, assuming that the volume of 1 g of saliva is equivalent to 1 g, and the salivary flow rate was calculated based on the weight/volume of saliva (mL) divided by the collection time (min). The unit is mL/min.

### Total protein content (TPC)

The total protein content in saliva was determined by the BCA method. According to the method described by Walker et al.^76^, 100 µL of saliva was added to a centrifuge tube, followed by 200 µL of working solution containing BCA, sodium tartrate, Na_2_CO_3_, NaOH, NaHCO_3_ and CuSO_4_·5H_2_O, incubated in a water bath at 60 °C for 30 min, cooled to room temperature, and 200 µL was added into 96-well plates The absorbance values at 562 nm were measured using calcium flowmeter. The concentration was estimated using BSA as standard.

### Salivary thiol/sulfhydryl (SH) content

For determination of salivary sulfhydryl concentration, the dithionitrobenzoic acid (DTNB) assay method was used with slight modifications. Saliva was diluted at a ratio of 1:3 with 0.25 mM Tri-HCl buffer solution (pH 8.3). The solution was kept at room temperature for 3 h to achieve maximum colour development after the addition of 50 µL of 10 mM DTNB, and absorbance was read at 412 nm, and the baseline values were subtracted. The thiol, or more accurately the SH, content was quantified using L-cysteine as a standard^77,78^.

### Antioxidant capacity (Ferric reducing antioxidant power, FRAP)

The method was slightly modified from the method described by Benzie et al.^79^. 200 µL of working solution was added to the 96-well plate, pre-warmed to 37 °C, and 20 µL of saliva sample was added to it. After incubation at room temperature for 4 min, the absorbance values at 593 nm were recorded using the 96-well ELISA. The final results were presented in terms of FeSO_4_ equivalent, and the antioxidant power of saliva was expressed as mM FeSO_4_ equivalent antioxidant power.

### Uric acid concentration (UA)

Chemical methods based on the reduction of alkaline phosphotungstate by uric acid are interfered by reducing compounds, including common metabolites and drugs or their breakdown products, in addition to the need for deproteinization^80^. Therefore, enzymatic methods were used to determine the uric acid content of saliva to circumvent these interfering factors. Uric acid is converted to allantoin and hydrogen peroxide by the enzyme allantoin. In the presence of peroxidase (POD), the hydrogen peroxide formed affects the oxidative coupling of 3,5-dichloro-2-hydroxybenzenesulfonic acid (DHBS) and 4-aminoantipyrine (4-AAP) to form a red quinonimine dye that produces a colour intensity proportional to the concentration of uric acid. 50 µL of saliva was added to 2 mL of working solution and incubated for 15 min at room temperature, as determined by measuring the increase in absorbance at 520 nm.

### Malondialdehyde content (MDA)

The method was slightly modified and described by Tarboush et al.^20^. 50 µL TEP standard solution, 50 µL saliva and 50 µL Tris-HCl buffer (as blank) were added to the centrifuge tubes, and 200 µL TCA solution and 200 µL TBA solution were added to each tube and mixed well. It was then boiled with water for 15 min, cooled in ice water bath to room temperature, and centrifuged at 1000 g for 10 min. 200 µL of supernatant was transferred into a 96-well plate and the absorbance at 532 nm was measured.

### Statistical analysis

XLSTAT Statistical software was used for statistical analysis. One-way analysis of variance (ANOVA) was used to compare significant differences in salivary parameters among stage 1 (before tea consumption), stage 2 (3-min after tea consumption) and stage 3 (30-min after tea consumption). Tukey post hoc analysis of variance was performed to observe the differences between groups. Probabilities less than 0.05 were considered statistically significant. After the value of saliva variable was converted into yield, principal component analysis (PCA) was used to analyse the variables at different stages. Pearson correlation tests the correlation between variables.

### Reporting summary

Further information on research design is available in the Nature Research Reporting Summary linked to this article.

### Supplementary information

Reporting Summary

## Data Availability

The authors declare that the data supporting the findings of this study are available within the article.
